# Analysis of Scattering by Plasmonic Gratings of Circular Nanorods Using Lattice Sums Technique

**DOI:** 10.3390/s19183923

**Published:** 2019-09-11

**Authors:** Vakhtang Jandieri, Kiyotoshi Yasumoto, Jaromir Pistora, Daniel Erni

**Affiliations:** 1 General and Theoretical Electrical Engineering (ATE), Faculty of Engineering, University of Duisburg-Essen, and CENIDE—Center for Nanointegration Duisburg-Essen, 47048 Duisburg, Germany; daniel.erni@uni-due.de; 2 Faculty of Information Science and Electrical Engineering, Kyushu University, Fukuoka 819-0395, Japan; kiyoyasumoto@kag.bbiq.jp; 3 Nanotechnology Centre, VSB-Technical University of Ostrava, 17. listopadu 15, 708 33 Ostrava—Poruba, Czech Republic; jaromir.pistora@vsb.cz

**Keywords:** periodic structures, plasmonics, refractive index sensors, scattering, bandgap structures

## Abstract

A self-contained formulation for analyzing electromagnetic scattering by a significant class of planar gratings composed of plasmonic nanorods, which were infinite length along their axes, is presented. The procedure for the lattice sums technique was implemented in a cylindrical harmonic expansion method based on the generalized reflection matrix approach for full-wave scattering analysis of plasmonic gratings. The method provided a high computational efficiency and can be considered as one of the best-suited numerical tools for the optimization of plasmonic sensors and plasmonic guiding devices both having a planar geometry. Although the proposed formalism can be applied to analyze a wide class of plasmonic gratings, three configurations were studied in the manuscript. Firstly, a multilayered grating of silver nanocylinders formed analogously to photonic crystals was considered. In the region far from the resonances of a single plasmonic nanocylinder, the structure showed similar properties compared to conventional photonic crystals. When one or a few nanorods were periodically removed from the original crystal, thus forming a crystal with defects, a new band was formed in the spectral responses because of the resonant tunneling through the defect layers. The rigorous formulation of plasmonic gratings with defects was proposed for the first time. Finally, a plasmonic planar grating of metal-coated dielectric nanorods coupled to the dielectric slab was investigated from the viewpoint of design of a refractive index sensor. Dual-absorption bands attributable to the excitation of the localized surface plasmons were studied, and the near field distributions were given in both absorption bands associated with the resonances on the upper and inner surfaces of a single metal-coated nanocylinder. Resonance in the second absorption band was sensitive to the refractive index of the background medium and could be useful for the design of refractive index sensors. Also analyzed was a phase-matching condition between the evanescent space-harmonics of the plasmonic grating and the guided modes inside the slab, leading to a strong coupling.

## 1. Introduction

With the rapid development of nanoscience and nanotechnology, the interaction of light with nanoscaled objects remains as an important issue in photonics because of their novel applications to sensors, imaging, and densely integrated devices [1,2,3,4,5,6,7]. Unique optical properties of metal nanostructures have become feasible because of the emergence of surface plasmons, which exist when the real part of permittivity of the metal is negative for the wavelength of the assumed optical excitation. Surface plasmon resonances create sharp spectral absorption and scattering peaks, as well as a strong electromagnetic near-field enhancement. Studies on the interaction of light with the plasmonic structures are organized in many different ways, being dependent on the dimensionality of the underlying objects and exciting sources. Review works about surface plasmon resonance sensors are presented in [8,9].

Scattering of an incident plane wave by plasmonic gratings composed of a periodic arrangement of metal and metal-coated dielectric nanocylinders was rigorously investigated utilizing our original formalism based on the lattice sums technique [10,11,12]. The method is briefly discussed in Section 2. Computational efficiency defines the main advantage of the developed formalism. Hence, it could be considered as a useful tool for designing and optimizing compact plasmonic devices tailored to advanced applications, such as in bio-analytics and/or medical diagnostics. The goal of the manuscript is to apply the proposed formalism to various configurations of plasmonic structures and demonstrate its usefulness from the viewpoint of flexible design, not only of plasmonic sensors, but also filters, reflectors, and guiding plasmonic devices. 

We should point out one important fact. The proposed method is self-contained and can easily be modified to analyze the modal fields and radiated fields in a wide class of planar plasmonic structures, thus representing a smart approach to functional plasmonic device design. Here, the developed procedure was implemented in FORTRAN and applied to the analysis of electromagnetic scattering encompassing three configurations of plasmonic structures; however, the class of plasmonic structures that can be analyzed using the proposed formalism is much broader than this. The first configuration was a multilayered periodic structure composed of cylindrical metal (silver) nanocylinders. Analogous to a conventional photonic crystal (PhC), it can inhibit electromagnetic wave propagation within a particular frequency range (i.e., a photonic bandgap). An understanding of the frequency bands, in which the electromagnetic wave propagation is suppressed, is an important issue in flexibly designing plasmonic guiding devices with relative low losses. The second configuration was formed by introducing periodic defects into the original PhC. The defects were introduced by periodically removing one or a few nanocylinders from a PhC layer. This approach gives us additional freedom to flexibly design the reflection and transmission bands as desired under the prescribed structural parameters, which is not possible for conventional configurations (structures without defects)**.** Our investigations showed that the periodic defects led to the appearance of a new band in the spectral responses due to the resonant tunneling through the defect layers. In order to analyze the gratings with periodic defects, a modification of the original formulation was needed and is briefly presented in the manuscript. The third configuration was a single array of metal-coated dielectric nanocylinders coupled to an isotropic dielectric slab and located in a background medium, whose refractive index was not equal to one. The structure was considered from the viewpoint of design of refractive index plasmonic sensors. 

## 2. Formulation of the Problem

**Figure 1:**
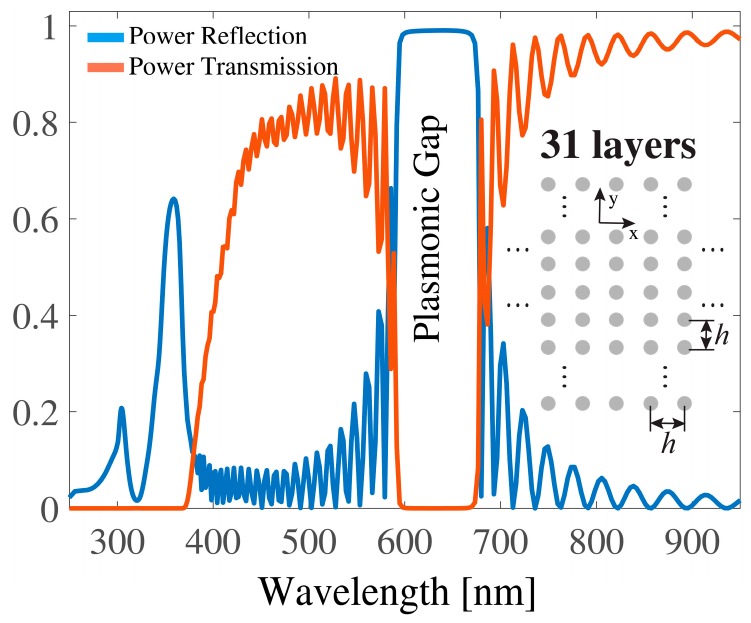
Power reflection (blue line) and power transmission (red line) of a 31-layered plasmonic crystal formed by the corresponding periodical arrangement of cylindrical Ag nanocylinders. Period of the grating was equal to *h* = 300 nm and radius of the nanocylinder amounts to *r* = 40 nm. Excitation was provided by an impinging H-polarized plane wave (*H*_z_, *E*_x_, *E*_y_) with normal incidence (along the positive *y*-axis).

**Figure 2:**
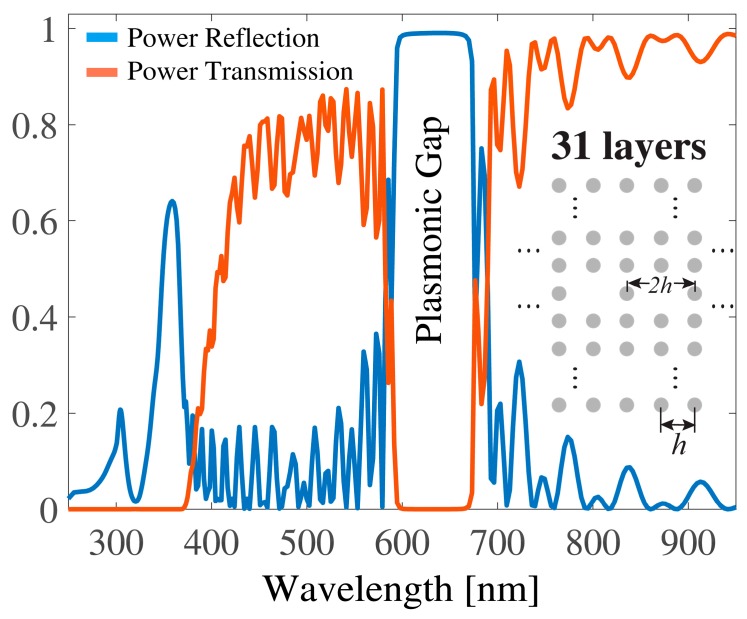
The same settings as in Figure 1, except that the intermediate 16th layer of the original crystal was replaced by a defect layer where one nanocylinder was periodically removed with a period of *2h*.

**Figure 3:**
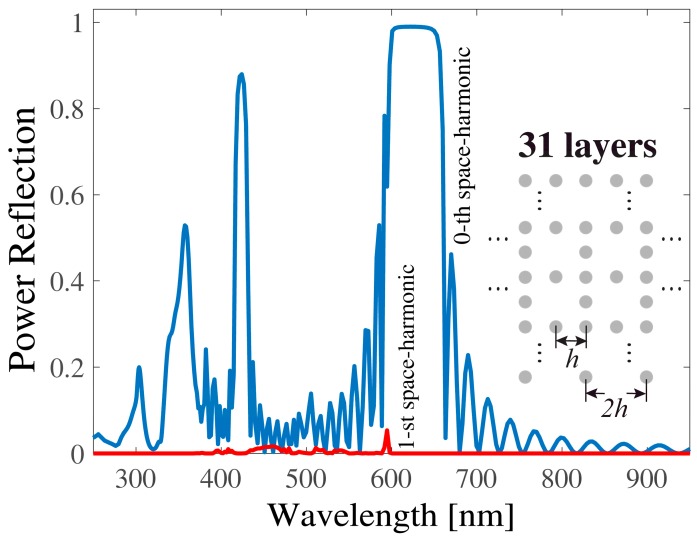
The same settings as in Figure 1, but the crystal was formed by alternately stacking the complete layer with a period *h* and the defect layer, where one nanocylinder was periodically removed according to the period *2h*.

**Figure 4:**
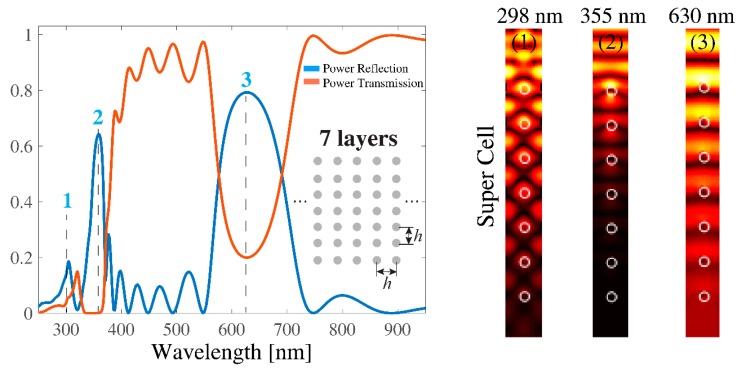
(**left**) Spectral response of the power reflection/transmission for the same configuration in Figure 1, but for seven layers along the *y*-axis. (**right**) Near field |*H_z_*| distributions at three different peak wavelengths, i.e. 298 nm (associated with the Rayleigh wavelength), 355 nm (associated with a resonance wavelength of a single silver nanocylinder), and 630 nm (associated with plasmonic gap in Figure 1).

In case of multilayered planar arrays of scatterers, as shown as insets of Figure 1, Figure 2, Figure 3 and Figure 4, the scattered space harmonics impinged on the neighboring arrays as new incident waves and were scattered into another set of space harmonics, which then impinged back on the original array. This describes the multiple scattering process between the planar arrays. The scattering process from each layer of the structure was characterized by the reflection and transmission matrices, which relate a set of the incident space harmonics to a set of reflected and transmitted ones. The incident and scattered fields were expressed as sets of cylindrical waves, and the reflection **R***_i_* and transmission **F***_i_* matrices for the arrays located on the *i*-th layer were given as follows [10,11,12]:

**R***_i_* = **U^+^**(*k_x_*_0_)[**I** − **T**(*k*_0_)**L**(*k_x_*_0_*h*, *k*_0_*h*)]^−1^**T**(*k*_0_)**P**(*k_x_*_0_), (1)

**F***_i_* = **I** + **U^−^**(*k_x_*_0_) [**I** − **T**(*k_x_*_0_)**L**(*k_x_*_0_*h*, *k*_0_*h*)]^−1^**T**(*k*_0_)**P**(*k_x_*_0_), (2)

with

(3) $$P\left(k_{x0}\right)\;=\left[P_{sq}\left(k_{x0}\right)\;\right]=\;\;\left[{\left(-j\right)}^{s}e^{-js\cos^{-1}\left(k_{xq}/k_{0}\right)}\right],\;$$

(4) $$U^{\pm }\left(k_{x0}\right)\;\;=\left[U_{ps}^{\pm }\;\left(k_{x0}\right)\right]=\;\;\left[\frac{2j^{s}}{k_{yp}h}e^{\mp js\cos^{-1}\left(k_{xp}/k_{0}\right)}\right],\;$$

(5) $$L\left(k_{x0}h,k_{0}h\right)=\left[L_{q-s}\left(k_{x0}h,k_{0}h\right)\;\right],$$

where *k*_0_ is the free-space wavenumber, the *p*-th space harmonic has an *x*-dependence as $e^{-jk_{xp}x}$, with a wavenumber *k_xp_* = *k_x_*_0_ + 2π*p*/*h* (orthogonal system and *h* parameter are specified in Figure 1). Here, **P**(*k_x_*_0_) labels the matrix that transforms the down-going *q*-th incident space harmonic wave to the *s*-th cylindrical harmonic wave. **U^+^** and **U^−^** are the matrices transforming the *s*-th cylindrical harmonic wave back to up-going and down-going *p*-th space-harmonic waves (with *p*, *s, q* = −*M*, −*M* +1, …, 0, …, *M* − 1, *M*). The *p*-th row and *q*-th column elements of the reflection $R_{i}\;\;=[R_{i}^{pq}]$ and transmission $F_{i}\;\;=[F_{i}^{pq}]$ matrices represent the reflection and transmission coefficients of the *p*-th order reflected and transmitted space harmonics for the *q*-th order space harmonic of the incident wave, respectively. Note that **I** denotes the unit matrix, **T**(*k*_0_) is the T-matrix of the isolated cylindrical rod [10] describing the nature of the scattered field per unit cell, and **L**(*k_x_*_0_*h*, *k*_0_*h*) is called the lattice sums. The lattice sums **L**(*k_x_*_0_*h*, *k*_0_*h*) in Equation (5) characterize the periodic arrangements of the scatterers and are independent of the polarization, of their observation points, and geometrical parameters. The convergent and very efficient calculation of the lattice sums for complex values of *k_x_*_0_ (in the modal analysis) were demonstrated in our recent work [12]. The situation was slightly different when we had a single periodic layer. In this case, we were not dealing with the reflection and transmission matrices, but with the reflection and transmission vectors. This was because we did not have any multiple interaction between the layers, and **P** in Equation (3) is not a matrix, but rather a vector. Finally, since the reflection and transmission matrices for single arrays were defined, the generalized reflection matrix viewed from the region of plane wave incidence could be easily obtained based on a recursive algorithm [10,13]. The calculation process was very fast, since only simple matrix multiplications were involved.

Furthermore, the method can be extended to scatterers with non-circular cross section, by numerically deriving the relevant T-matrix expression [14]. In case of a circular cylinder, the T-matrix has a quite simple form—it is a diagonal matrix—that can be very easily calculated in closed form. However, for some other non-circular cross sections, the T-matrix is not anymore diagonal and should be calculated numerically. 

## 3. Numerical Results and Discussions

In the numerical experiments, we assumed Ag (silver) for the metal [15], which is commonly used in plasmonics. It is worth mentioning that an important advantage of the proposed approach is its computation efficiency. The desktop CPU runtime on the 3.6 GHz Intel Core i7 with 8 GB RAM per one frequency point was approximately 0.03–0.05 s for all three configurations shown below. 

### 3.1. Light Scattering by Multlayered Structures of Plasmonic Nanocylinders with and without Defects

Periodic defects were introduced by periodically removing one or several rods from the layer. The Equations (1)–(5) cannot be directly used for the arrays having different periods. A modification of Equations (1)–(5) was needed. Without loss of generality, let us assume that the structure is composed of arrays having periods *h* and 2*h* (the layer with a period 2*h* is formed by periodically removing one nanocylinder). The problem can be solved by decomposing the original set of space harmonics with the period 2*h* into subsets of space harmonics with a period *h.* In other words, for the arrays with the period *h*, we introduced two subsets of space harmonics as follows [16]:

(6) $$\left\{e^{jk_{xp}x};\;\;k_{xp}=k_{x0}\;+\;2\pi p/2h\right\}=\overset{1}{\underset{\nu =0}{\cup }}\;\left\{e^{j\left(\xi_{\nu }+\frac{2\pi p}{h}\right)x};\;\;\xi_{\nu }=k_{x0}+\nu \pi /h\right\}.\;$$

Taking into account the orthogonal nature of the space harmonics, the reflection and transmission matrices for the arrays with the period *h* can be calculated using the similar expressions of Equations (1)–(5), but under the incidence of two different subsets of space harmonics. The results were then rearranged on the basis of the original set of space harmonics with the period 2*h*. For the arrays with the period *h*, we used two subsets of space harmonics with the wavenumbers $\xi_{0}$ and $\xi_{1}$, as shown in Equation (6). The generalization of the problem for a wide class of structures with periodic defects is straightforward.

In Figure 1, Figure 2 and Figure 3, multilayered structures having a period *h* = 300 nm and composed of Ag nanocylinders with a radius *r* = 40 nm are numerically investigated. Without loss of generality, 31 layers were considered and the background medium was a free space. Usually, a “planar plasmonic structure” means that its thickness is not larger than several hundred nanometers. Our structure probably was not the best example for the planar geometry; however, the wide plasmonic gap could be observed for the multilayered structure and thus it is a reason to consider this configuration. Nonetheless, our formalism can be easily applied to various planar configurations such as the second example in the manuscript. We discuss the frequency response of the power reflection and power transmission under normal incidence of an exciting plane wave. The normal incidence is the usual configuration of the plasmonic crystal for the use as a frequency and polarization selective device. All numerical results were carried out with an error measure in the energy conservation of less than $10^{-7}$ [17]. For better understanding the impact of periodic defects on the spectral responses, we considered three configurations, as illustrated as insets in Figure 1, Figure 2 and Figure 3. Figure 1 displays the original configuration with no defect included. It consisted of a square lattice formed by cylindrical Ag nanocylinders. In Figure 2, only the intermediate layer (the 16th layer) of plasmonic crystal was replaced by a defect layer of the lattice, in which one cylinder was periodically removed, and thus a layer with a period 2*h* was formed. In Figure 3, the plasmonic crystal was generated by alternately stacking a complete layer and a defect layer where one nanocylinder was accordingly removed. The defects were both periodic in the *x*- and *y*-direction with a period 2*h*. The spectral response of both the power reflection (blue line) and the power transmission (red line) for the incidence of (*H*_z_, *E*_x_, *E*_y_) wave are plotted in Figure 1. From the spectra, it can be seen that an original plasmonic crystal had a wide reflection band (plasmonic gap) within the visible wavelength region 600 nm < λ < 680 nm, which was far from the plasmon resonance of the individual plasmonic nanocylinder at 355 nm. Note that a separation distance between the nanocylinders along the transverse *y*-direction was quite large *h* > 2*r* and, therefore, no coupling occurred between localized plasmons on the metal nanocylinders. It could be seen that the transmission was suppressed within a short wavelength band (250 nm < λ < 380 nm). When the 16th layer of the lattice was replaced by a defect layer, as shown in Figure 2, the wide reflection band was barely affected by the defects and retained its original bandwidth. When the defect layers were periodically assembled in the *y*-direction, as shown in Figure 3, the frequency response of the reflection was significantly modified compared to those of Figure 1 and Figure 2. As shown in Figure 3, an additional narrow band was formed in the shorter wavelength region around 425 nm because of the resonant tunneling through the defect layers. Since the period of the lattice along the *x*-direction was 2*h*, the first space harmonic began to propagate within the indicated wavelength range, with the spectral response of its amplitude also being marked by a red line.

Next, for comparison, we study the seven-layered structure composed of Ag nanocylinders, whilst the other geometrical parameters were the same as those already used in Figure 1. The corresponding spectral responses of the power reflection and power transmission coefficients are plotted in Figure 4 by blue and red lines, respectively. The near field distributions within the plasmonic lattice’s super cell were calculated at three different wavelengths, namely at 298 nm (marked by “1”), at 355 nm (marked by “2”), and at 630 nm (marked by “3”). A strong reflected field was observed at 298 nm, associated with the Rayleigh wavelength (λ ≈ *h/p* at the normal incidence, where *p* denotes the space-harmonic), whereas the field was enhanced at the surface of the uppermost nanocylinder layer at *λ* = 355 nm. The latter was related to the interaction of the incident wave and the uppermost individual nanocylinder. Unlike Figure 1, the strong plasmonic gap was not formed in case of seven layers, and therefore the near field distribution at *λ* = 630 nm showed only a partially reflected field from the multilayered periodic structure. Our analysis showed that at least 13 layers were needed to realize a plasmonic gap that fully evolved to the one shown in Figure 1 (only when others parameters were fixed).

### 3.2. Coupling between Plasmonic Grating and a Dielectric Slab for Application as a Refractive Index Sensor 

**Figure 5:**
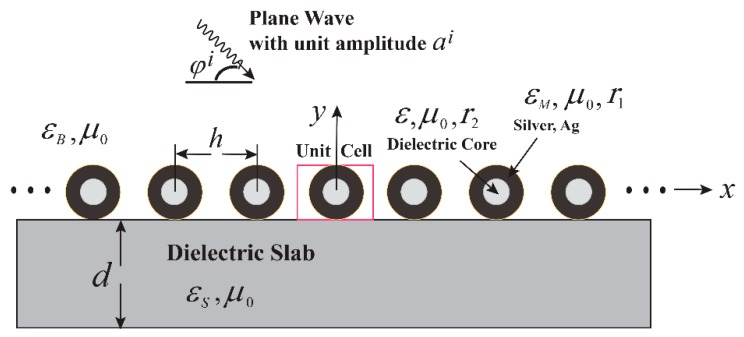
Cross-sectional view of the plasmonic grating composed of Ag-coated dielectric nanocylinders periodically arranged on a dielectric slab with thickness *d*. Material parameters of the background medium, the coating metal, the dielectric core, and dielectric substrate were $\left(\varepsilon_{B},\;\mu_{0}\right)$, $\left(\varepsilon_{M},\;\mu_{0},\;r_{1}\right)$, $\left(\varepsilon ,\;\mu_{0},\;r_{2}\right)$, and $\left(\varepsilon_{s},\;\mu_{0}\right)$, respectively. The excitation was provided by an impinging *H*-polarized plane wave with an incident angle $\varphi^{i}$.

**Figure 6:**
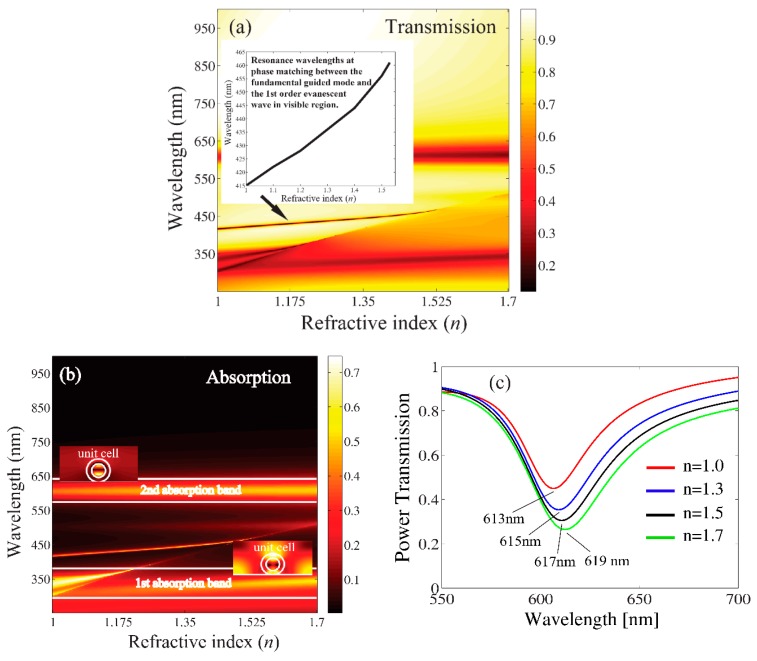
Plasmonic grating as refractive index sensor: Contour plots of transmission spectra (**a**) and absorption spectra (**b**) versus refractive index $n=\sqrt{\varepsilon_{B}/\varepsilon_{0}}$ of the background medium (Figure 4) at $r_{1}$ = 40 nm, $r_{2}$ = 20 nm, *h* = 300 nm, $\varepsilon /\varepsilon_{0}=6.5$, *d* = 200 nm, $\varepsilon_{s}/\varepsilon_{0}=2.5$ and $\varphi^{i}=90{}^{\circ}$. The regions of the first and second absorption bands are marked by the white lines. Power transmission spectra in the second absorption band for four different values of the refractive index of the background medium (**c**).

We studied Ag-coated dielectric nanocylinders with the following structural parameters: $r_{1}=40$ nm, $r_{2}=20$ nm, *h* = 300 nm, $\varepsilon /\varepsilon_{0}=6.5$, *d* = 200 nm, and $\varepsilon_{s}/\varepsilon_{0}=2.5$ (glass). Note that in the first part, the pure Ag nanocylinders were considered, and in the second part, the Ag-coated elements were considered. For a metal-coated nanocylinder, special attention was paid to the appearance of “the second absorption band” (Figure 6), which was never observed for a single pure metal rod and was found to be very sensitive to the refractive index of the background medium. The geometry of the problem is illustrated in Figure 5. The refractive index of the background medium was *n*. The structure can be used for the design of refractive index plasmonic sensors. Figure 6a shows the contour plots for the power transmission of the 0-th diffraction order and the absorption coefficients as a function of the wavelength within the range of 250 nm < λ < 1000 nm and the refractive index 1≤n≤1.7 at normal incidence $\left(\varphi^{i}=90{}^{\circ}\right)$. In this range of refractive indices, the background medium can, for example, be considered as a fluid, such as water, chloroform, benzene, or carbon disulfide. Figure 6b demonstrates two absorption bands attributable to the excitation of localized surface plasmons. The first absorption band, 300 nm < λ < 380 nm, was attributable to the surface plasmons supported by the outer interface of the metal coating resonating with the incident wave, whereas the second absorption band, 550 nm < λ < 650 nm, was formed because of the excitation of the surface plasmons along the boundary layer between the metal and the inner dielectric core. For better understanding of the problem, we also numerically studied the near field distributions of the magnetic field $\left|H_{z}^{}\right|$ within these absorption bands, and the enlarged figures of the super cell are shown as inset in Figure 6b. From these figures, it can be noted that in the first absorption band region the resulting surface field was predominantly oriented towards the illumination side of the nanocylinders. There was almost no field visible inside the dielectric core of the nanocylinder, whereas a strong field enhancement along the surface between the metal and the inner dielectric core region was visible in the region of the second absorption band (only for a coated nanorod). Figure 6c depicts the transmission spectra in the wavelength range, 550 nm < λ < 650 nm (second absorption band), for four different values of the refractive index of the background medium. It can be observed that the resonance wavelengths sensitively depended on the refractive index and were thus shifted to longer wavelengths with an increasing index of refraction *n*, which could be exploited in the context of a refractive index sensor.

**Figure 7:**
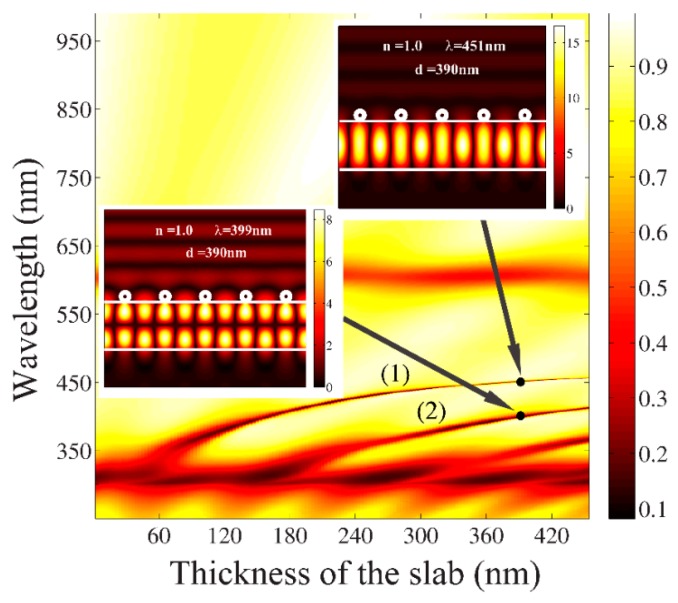
Plasmonic grating as refractive index sensor: Contour plots of transmission spectra versus thickness of the slab *d* at *n* = 1. Simulated near field distributions of $\left|H_{z}\right|$ at λ = 451 nm and λ = 399 nm for a fixed thickness of the slab *d* = 390 nm are shown as insets.

Another important issue in the application of refractive index sensing concerned the coupling mechanism between the plasmonic grating and the underlying dielectric slab. When the phase matching condition [17,18],

(7) $$\frac{\lambda }{d}\;\frac{1}{\pi \sqrt{\varepsilon_{s}-{\left(\frac{\beta \lambda }{2\pi }\right)}^{2}}}\left[\tan^{-1}\left(\frac{\varepsilon_{s}}{\varepsilon_{B}}\sqrt{\frac{{\left(\frac{\beta \lambda }{2\pi }\right)}^{2}-\varepsilon_{B}}{\varepsilon_{s}-{\left(\frac{\beta \lambda }{2\pi }\right)}^{2}}}\right)+m\frac{\pi }{2}\right]=1$$

(8) $$\beta =\frac{2\pi }{h}p\;(p=\pm 1,\;\pm 2,\;\pm 3,\;\cdots ),$$

between the guided slab mode *m* and one of the *p*-th space-harmonics was fulfilled at some specific wavelength, these two wave components effectively interacted and a fraction of the power of the incident wave was resonantly transferred to the guided wave along the slab waveguide. It led to a substantial decrease of the transmitted power at these particular wavelengths (400 nm < λ < 470 nm), as depicted in Figure 6a. In this wavelength range and under our prescribed geometrical parameters, a phase matching between the fundamental *H*-polarized slab mode and the scattered wave of the first diffraction orders was attained. We estimated the sensitivity *S* by monitoring the wavelength shift of the transmission peak from $\lambda_{air}$ (resonance wavelength at the coupling between the plasmonic grating and the slab in the air) to $\lambda_{B}$ (resonance wavelength at coupling between the plasmonic grating and the slab in the background medium) as S=Δλ/Δn = $\left(\lambda_{B}-\lambda_{air})/(n-1\right)$. At 1 < *n <* 1.4, the sensitivity was equal to 72, and at *n >* 1.4, the sensitivity was 84. When *n^2^* was close to permittivity $\varepsilon_{s}$ of the dielectric slab, the field confinement inside the slab became weaker and the dielectric slab lacked in supporting any guided modes. Hence, no guided mode resonance was observed in the transmission spectra at *n >* 1.58, as depicted in Figure 6a. In order to provide a deeper insight in the formation of the resonance due to the coupling between the grating and the slab, we investigated the dependence of the transmission spectra versus the thickness of the slab *d* when the background was a free space. Two regions of the resonance peaks showed an almost linear behavior, marked as Equations (7) and (8), and are displayed in Figure 7. The first one represented the resonance peak attributable to the coupling of the first evanescent wave (the first space-harmonic) with the fundamental *m* = 0 guided mode, whereas the second one demonstrated the resonances attributable to a coupling of the first evanescent wave with the *m* = 1 guided mode inside the slab. The simulated near field distributions at *λ* = 399 nm (associated with the coupling of the first evanescent wave of the grating with the *m* = 1 guided mode) and *λ* = 451 nm (associated with the coupling of the first evanescent wave of the grating with the *m* = 0 guided mode) for a fixed slab thickness *d* = 390 nm are shown as insets in Figure 7. Our analysis showed that these sharp resonances were sensitive to the background refractive index and can be considered as one of the components for a design of refractive index sensors. Finally, we noted that the sensor characteristics shown in the manuscript needed some improvements. We hope that the fast and rigorous numerical method we developed will help engineers and researchers to effectively analyze a wide class of plasmonic gratings in order to design very highly sensitive plasmonic sensors.

## 4. Concluding Remarks

A rigorous self-consistent formulation was efficiently applied to a wide class of plasmonic gratings composed of metal and metal-coated nanocylinders. In particular, scattering by multilayered plasmonic crystals with and without defects was analyzed. The coupling mechanism of the plasmonic grating with the dielectric slab was studied from the viewpoint of application as a refractive index sensor. The method was computationally very fast and thus represents an effective tool that is apt for designing and optimizing tailored sensors (particularly with optimized sensitivity), such as for advanced biomedical applications. The proposed formalism can also be applied to analyze the interaction of the planar plasmonic grating with non-isotropic media, such as, for example, a magneto-optical [18] or magnetized ferrite [19] slab. Generalization of the described method towards the multilayered sandwiched structures was straightforward. Such a combination enables one to achieve a multiple increase in sensitivity when compared to a sensor structure having just one metal grating (or layer). Another point worth noticing is that our formalism can be easily applied to multilayered metal-dielectric plasmonic nanocylinders with eccentric configuration per unit cell. Eccentric configurations of metal-dielectric plasmonic nanocylinders are being investigated in the framework of emerging Fano resonances [20].
